# Supplementary material for: Plasma BDNF concentrations and the antidepressant effects of six ketamine infusions in unipolar and bipolar depression
Source: PeerJ. 2021 Mar 29;9:e10989. doi: 10.7717/peerj.10989 (PMC8015784; doi:10.7717/peerj.10989)
Supplement: Supplemental Information 2 [file peerj-09-10989-s002.docx]

Supplemental Table 1. Comparison of MADRS scores and pBDNF concentrations between responders and nonresponders and between remitters and nonremitters in patients with TRD using linear mixed model analysis.

| Outcomes | Variables | Group-by-time interaction | | Time main effect | | Group main effect | |
| --- | --- | --- | --- | --- | --- | --- | --- |
|  |  | F | *p* | F | *p* | F | *p* |
| Responders vs. nonresponders | MADRS scores | 52.67 | **<0.001** | 182.13 | **<0.001** | 45.78 | **<0.001** |
|  | pBDNF concentrations | 0.49 | 0.484 | 8.63 | **<0.001** | 2.21 | 0.116 |
| Remitters vs. nonremitters | MADRS scores | 71.77 | **<0.001** | 213.16 | **<0.001** | 20.86 | **<0.001** |
|  | pBDNF concentrations | 0.50 | 0.480 | 7.26 | **0.001** | 1.49 | 0.232 |
| Bolded values are *p*<0.05.  Abbreviations: pBDNF=plasma brain derived neurotrophic factor; MADRS=the Montgomery-Asberg Depression Rating Scale; TRD=treatment refractory depression. | | | | | | | |

**Supplemental Table 2.** Correlation of baseline pBDNF concentrations and MADRS scores at 13 d or 26 d in patients with TRD.

| Variables | MADRS scores at 13 d | MADRS scores at 26 d |
| --- | --- | --- |
| Baseline pBDNF concentrations (ng/ml) | *r*=-0.299 | *r*=-0.377 |
|  | ***p*=0.008** | ***p*=0.003** |
| Bolded values are *p*<0.05.  Abbreviations: pBDNF=plasma brain derived neurotrophic factor; MADRS=the Montgomery-Asberg Depression Rating Scale; *r*=Pearson coefficient of correlation; TRD=treatment refractory depression. | | |

**Supplemental Figure 1.** Change in depressive symptoms in patients with TRD.

^
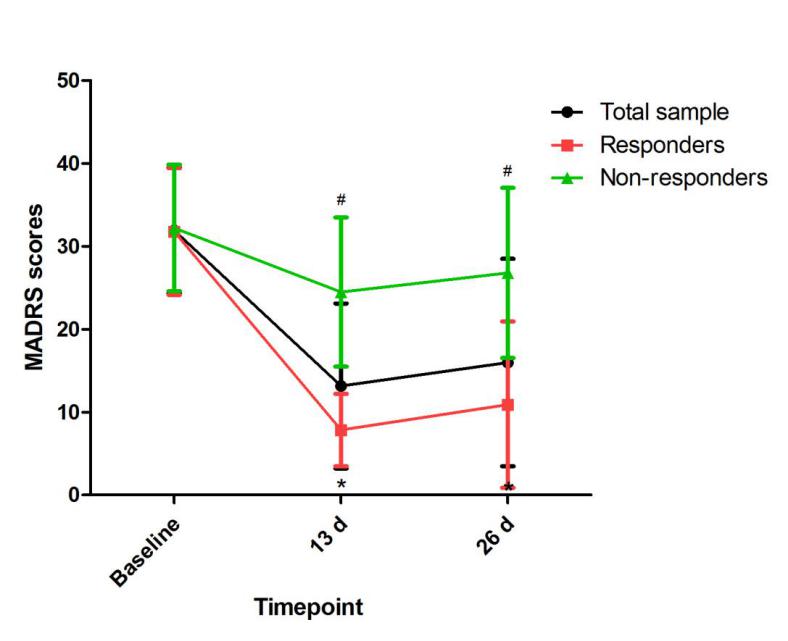

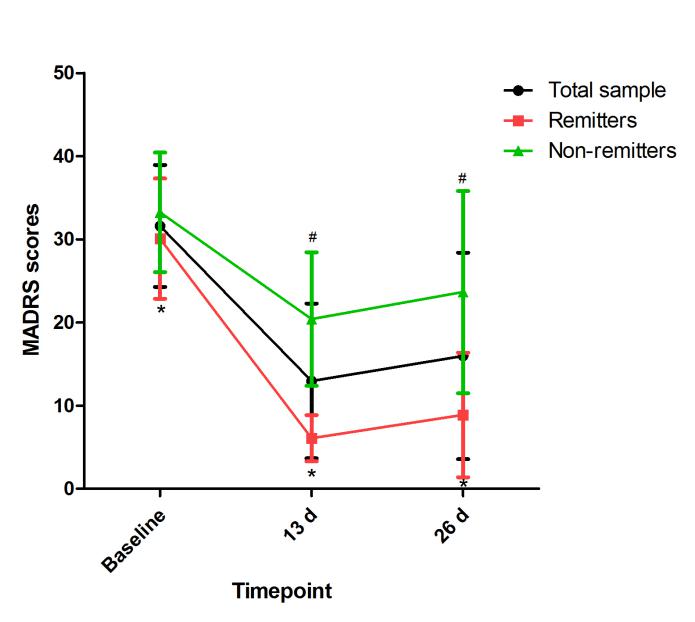
^

^#^Significant difference was found when comparing baseline to the indicated times (*p<*0.05).

^*^Significant difference was found between responders and nonresponders and between remitters and nonremitters at the indicated times (*p<*0.05).

Abbreviations: MADRS=the Montgomery-Asberg Depression Rating Scale; TRD=treatment refractory depression.

**
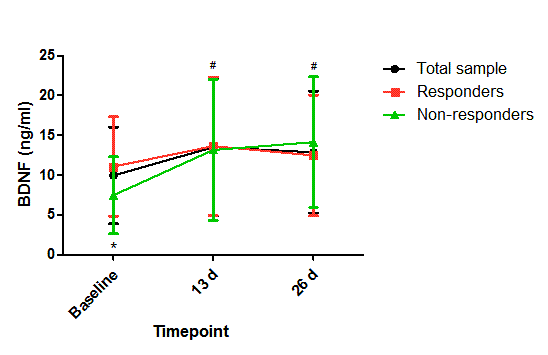

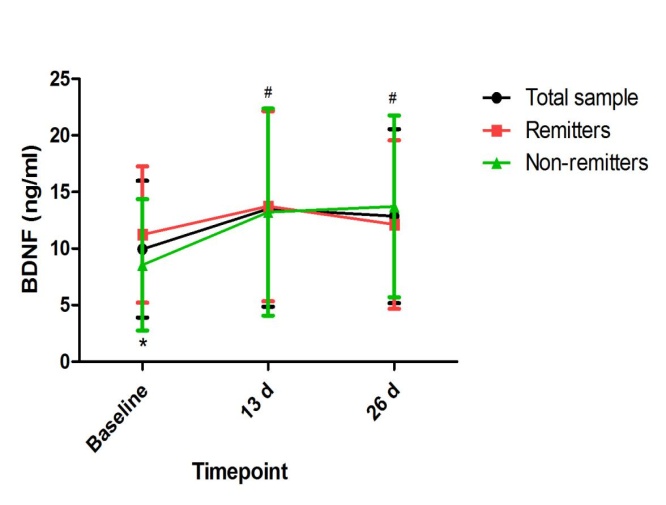
Supplemental Figure 2.** Change in pBDNF concentrations in patients with TRD.

^#^Significant difference was found when comparing baseline to the indicated times (*p<*0.05).

^*^Significant difference was found between responders and nonresponders and between remitters and nonremitters at the indicated times (*p*<0.05).

Abbreviations: pBDNF=plasma brain derived neurotrophic factor; TRD=treatment refractory depression.
