# Supplementary material for: Plasma BDNF concentrations and the antidepressant effects of six ketamine infusions in unipolar and bipolar depression
Source: PeerJ. 2021 Mar 29;9:e10989. doi: 10.7717/peerj.10989 (PMC8015784; doi:10.7717/peerj.10989)
Supplement: Supplemental Information 3 [file peerj-09-10989-s003.docx]

**Objectives of Study：**

(1) To determine the antidepressant efficay of ketamine in treatment-resistant depression (TRD);

(2) To sequencing RNA of patients who absorb in TRD with ketamine treatment, and find out genes which change its expression after ketamine treatment;

(3) To explore the feasibility of ketamine treatment in TRD, and find out related genes and other blood samples of antidepressant efficacy and side-effect of this treatment.

**Inclusion criteria:**

(1) Male or female patients, 18-65 years of age;
(2) Treatment-resistant depression that meet the DSM-5 diagnostic criteria, including unipolar and bipolar depression: patients were treated ineffectively by two kinds of antidepressants with sufficient dosage and full period course of treatment. Sufficient dosage means the maximum doses of more than two-thirds (equal to imipramine doses of 150~200mg/d), full period of treatment refers to adequate treatment for more than 4 weeks. Patients who are adsorbed in chronic depression must be treated more than 8 weeks. Ineffectiveness means score-reducing rate of Hamilton Depression Scale < 30%; or with apparent suicidal ideation as measured by the Beck Scale for?Suicide?Ideation;
(3) Scores of Hamilton Depression Scale must over 17 when participants join in this study initially;
(4) Participants must have junior middle school and above years of schooling;
(5) Each participant must join in the study voluntarily and sign an informed consent.

**Exclusion criteria：**

(1) Other major psychiatric disorders that meet the DSM-5 diagnostic criteria include organic mental disorders, alcohol dependence, drug dependence/abuse, schizophrenia, etc;
(2) Currently, there are serious and unstable somatic diseases such as diabetes, thyroid disease, hypertension, heart disease and narrow-angle glaucoma, etc;
(3) A history of neurological diseases such as epilepsy and dementia;
(4) Negative result on urotoxicological screening;
(5) Women who are at the stage of pre-pregnancy, pregnancy or lataction;
(6) Disobedient patients.

**Description for medicine or protocol of treatment in detail：**

(1) Patients are fasting eight hours before treatment; (2) Start monitoring body temperature, blood pressure, pulse and breathing at least 3 hours before treatment; (3) Infuse ketamine by infusion pump over 40 minutes. Treatment time was uniformly in the morning, 3 times per week, and 6 times during the two weeks; (4) Fasting during the injection of ketamine and 2 hours after injection; (5) Electrocardiogram monitoring are used to ensure the patient's safety during the injection of ketamine and 2 hours after injection.

The detailed trial protocol, please see <http://www.chictr.org.cn/showproj.aspx?proj=20875> (the registration number: ChicCTR-OOC-17012239)
